# Application of a Self-developed, Low-budget Indocyanine Green Camera in Surgical Imaging – a Single Institution’s Experiences

**DOI:** 10.1007/s10895-023-03224-0

**Published:** 2023-03-29

**Authors:** Zoltan Sandor, Zoltan Ujfalusi, Adam Varga

**Affiliations:** 1 Department of Surgery, University of Pécs, Pécs, Hungary; 2 Department of Biophysics, University of Pécs, Pécs, Hungary

**Keywords:** indocyanine green, fluorescence, surgery, development

## Abstract

Introduction: Indocyanine green is a fluorescent dye, the use of which is becoming more and more widespread in different areas of surgery. Several international studies deal with the dye’s usefulness in intraoperative angiography, the localization of tumors, the more precise identification of anatomical structures, the detection of lymph nodes and lymph ducts, etc. The application of the dye is safe, but a suitable equipment park is required for its use, which entails relatively high costs.

Objectives: The aim of our research is to create a detector system on a low budget, to be used safely in everyday practice and to illustrate its operation with practical examples at our own institute.

Methods: By modifying a web camera, using filter lenses and special LEDs, we created a device suitable for exciting and detecting indocyanine green fluorescence. We prove its excellent versatility during the following procedures at our institute: breast tumor surgery, kidney transplantation, bowel resection, parathyroid surgery and liver tumor resection.

Results: The finished camera has an LED light source with a peak wavelength of 780 nm, and the incoming light is filtered by a bandpass filter with a center wavelength of 832 nm. A low budget ($112), easy-to-use tool was created, which is suitable for taking advantage of the opportunities provided by indocyanine green.

## Introduction

Indocyanine green (ICG) is a special fluorescent dye, the excitation of which requires light with a wavelength between 600 and 900 nm with a peak of 780–800 nm. Under near-infrared illumination, it emits fluorescent light with a peak wavelength of about 830 nm in human blood [1]. It’s medical use is wide-ranging, as it is suitable for angiographic examination either intravenously or by other modalities and can be used in many areas of surgery. By examining the perfusion of organs, the safety of operations is increased, the detection of liver tumors becomes easier, through staining the lymphatic vessels, it is suitable for locating sentinel lymph nodes in breast tumor surgery, etc. It is safe and simple to use, has an extremely favorable side effect profile, and is quickly excreted into bile via the liver. Allergic reactions are reported in literature but are extremely rare. An impediment to its use is that both the excitation and the emitted light are invisible to the human eye, requiring a special lighting and detector system [2]. There are many devices on the market, the price of simple manual models starting from $2,000-$3,000. Among the dyes used for the tests, Verdye (Manufacturer: Diagnostic Green GMBH, Germany) is widespread, which is available at a price of about $100/ampoule.

The goal of our research is to create an illumination and detector system that can greatly reduce the cost of ICG programs without significant compromise in image quality and clinical usability.

## Introducing Our Own Tool

**Fig. 1:**
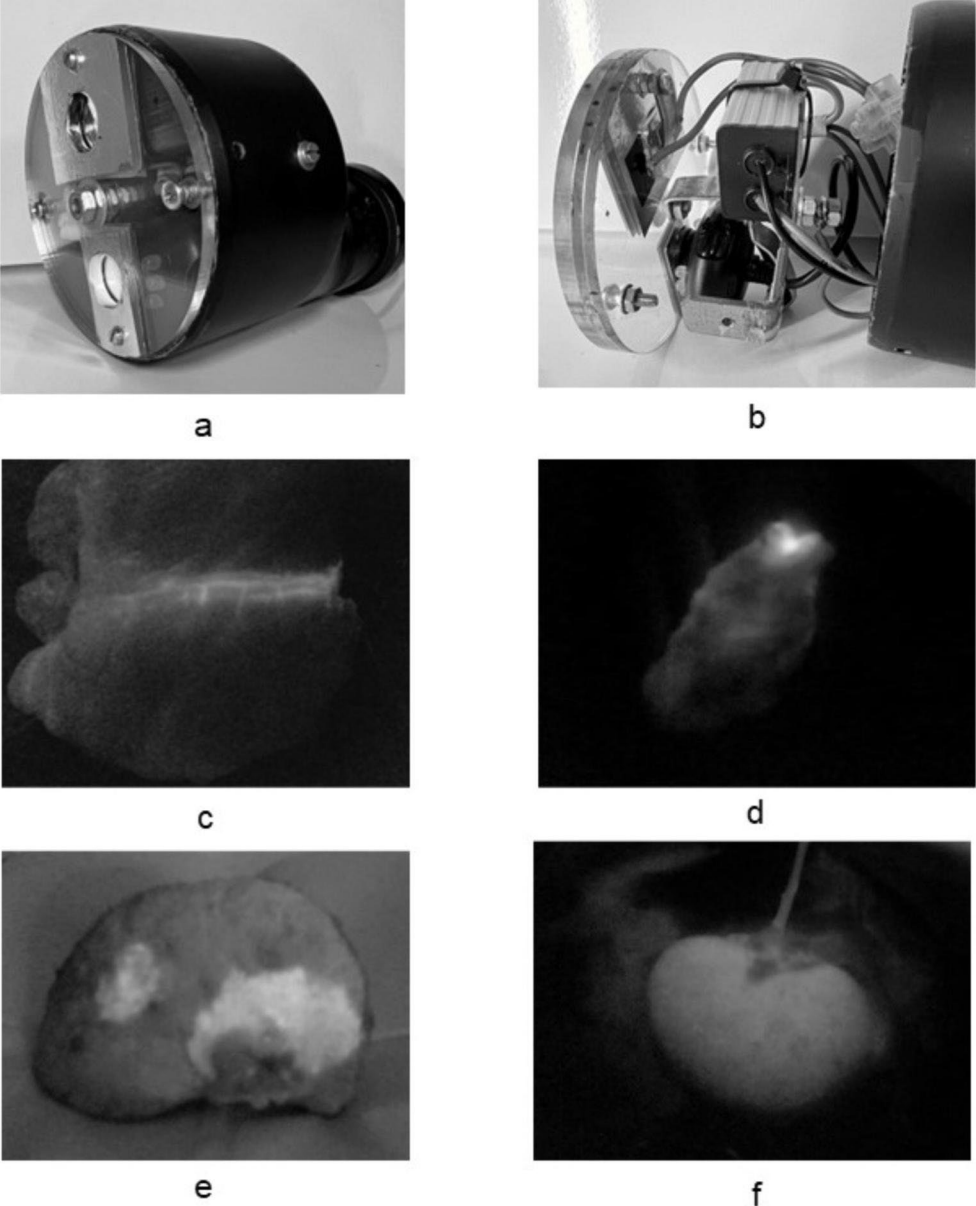
**a**: external image of our device. **b**: without the outer cover of our device. **c**: our first, experimental study, coronary staining of the pig heart (ICG is marked with white). **d**: breast tissue, white marking in the sentinel lymph node. **e**: liver tissue, white marking in the tumor. **f**: kidney perfusion test before transplantation

We based the design on the excitation and emission spectrum of ICG. Our camera is a Trust Spotlight Webcam Pro, VGA webcam (Trust International B.V., The Netherlands) (price: $13), from which the infrared filter has been removed. For the lighting, we purchased a 10 W near-infrared LED from eBay, which emits light with a wavelength of 780 nm (price: $18), as well as a 10 W LED driver for this LED (price: $3). We placed a special filter in front of the camera with the following parameters: center wavelength 832 nm, 10 nm width bandpass filter. Using the filter, only ICG fluorescence becomes visible. We also placed a filter in front of the LED, which filters out possible different wavelengths: center wavelength 770 nm, bandpass filter. The filters were purchased from Alibaba for $16 and $22, respectively. The system was assembled in a plastic housing with a control unit as shown in the picture, the assembly costs were about $40. (Fig. 1-a,b) The total cost of the camera was thus 112$. For the display, we used our own laptop, to which the webcam was connected with a USB cable. Due to its size and weight, the camera can easily be held in hand and directed to the target area even without a special prop. We used Verdye dye for the experiments and procedures.

## Our Tests with the Camera

### Experimental Investigation

After we were convinced that our device worked, we tested its usability on a pig’s heart for the first time. A coronary artery of a commercially available porcine heart was cannulated and then filled with ICG solution. With the help of our camera, the ICG filled vessel could be visualized perfectly. (Fig. 1-c)

### Breast Surgery

During breast tumor surgery, the identification and removal of the sentinel lymph node for histological examination is a critical issue. In practice, blue dye and isotopic labeling are used for this. In recent years, the use of ICG for the detection of lymphatic vessels and lymph nodes has become widespread [3]. During our study, a 50-year-old female patient underwent surgery for a breast tumor (ductal in situ carcinoma). To mark the sentinel lymph nodes, the surgeon routinely used double marking (blue dye + 99mTc radiopharmaceutical), which was supplemented with 10 mg ICG administered next to the areola immediately before the operation. During the breast excision and sentinel lymph node biopsy, the lymphatic vessels leading to the axillary region could be easily followed with the help of our camera and the lymph nodes could also be identified. After the operation, the specimen was also examined separately, where we found that a lymph node that did not accumulate either blue dye or the isotope was also stained with ICG. (Fig. 1-d) Later, our results were confirmed by the histological examination.

### Liver Surgery

There are several different modalities for the use of ICG during liver surgeries. Tumorous lesions of the liver accumulate ICG as a positive contrast agent. For this, it is necessary to administer the dye intravenously to the patient at least one day before the operation. In our own operation, a 54-year-old male patient operated on for hepatocellular carcinoma was given 25 mg of ICG intravenously the day before the operation, according to literature data. The following day, during the open liver surgery, in addition to the intraoperative ultrasound examination, we were also able to clearly identify the tumorous liver with our own camera. We could detect a fluorescent ring around the tumor, which is a typical sign for liver tumors. (Fig. 1-e) During examination of the specimen, the non-fluorescent liver edge could also be reliably identified; the later histological examination also confirmed tumor-free resection margins. The other option that is used for anatomical resections is where we actually perform angiography with ICG administered during surgery. The part of the liver to be resected is excluded from the circulation even before the administration of ICG, and thus the part of the liver perfused and not perfused with ICG becomes clearly visible, i.e. the resection border [4].

### Examination of Intestinal Perfusion

Due to the initial successes, our camera was also used in emergency surgeries. It was known to us from the literature that ICG angiography may help in the more accurate detection of bowel necrosis [5]. A 29-year-old female patient with type 1 diabetes and severe vasculitis affecting the abdominal blood vessels had to undergo acute surgery due to extensive intestinal necrosis. Intravenously administered 20 mg ICG during the exploration clearly outlined the patchy loss of circulation affecting the entire small intestine section, which had already led to definitive intestinal necrosis in several places. However, with the help of ICG angiography, the lack of circulation could be clearly seen even in areas that looked macroscopically healthy to the naked eye. The ICG findings also confirmed the fact that surgical therapy was no longer possible for the patient.

### Parathyroid Surgery

One area where ICG fluorescence has been most extensively researched is parathyroid surgery. During surgeries, it is often difficult to identify small parathyroid glands that are not clearly separated from their surroundings. During the operation, a few seconds after the administration of ICG, a fluorescent “flash” of the parathyroid glands is observed, which can help in identification [6]. We tested our camera during several parathyroid surgeries due to hyperparathyroidism and registered good results. With this technique, it is important to administer the ICG intravenously before the parathyroid gland is completely dissected, so that it may easily reach the parathyroid gland due to the intact blood supply. In all cases, the detection was performed after the administration of a dose of 5 mg of ICG intravenously.

### Examination of the Kidney before Transplantation

Adequate blood perfusion of the transplanted kidney is of particular importance during kidney transplants. During organ donation, problems with perfusion may arise, especially in the case of multiple renal arteries. ICG is suitable for checking adequate perfusion before transplantation and can also predict a delayed graft function. In addition to all this, it is also an excellent opportunity to map the blood supply of the ureter [7]. With our own camera, before a kidney transplant, during the back table preparation phase, we injected a minimal amount (2.5 mg) of Verdye into the one-liter perfusion solution. The kidney was perfused with the solution prepared in this way, and the perfusion of the entire organ and the tissues around the ureter were also examined. Based on the obtained image the perfusion of the kidneys that are about to be transplanted can be mapped excellently. (Fig. 1-f)

## Discussion

The use of indocyanine green dye has opened new paths in surgery, especially intraoperatively, in the field of real-time guided surgery. In addition to anatomical navigation, it also facilitates the intraoperative decision-making of the surgeon.

With the help of ICG cholangiography used during one of the most common surgical interventions, laparoscopic cholecystectomy, the clear identification of extrahepatic bile ducts doubled. 80% of the surgeons who used the method believed that cholecystectomy is much safer with the use of ICG [8].

In the surgery of the gastrointestinal tract, the healing tendency of the anastomoses created after resection is largely determined by the proper circulation of the anastomosed intestinal ends. Detection of perfusion and anastomotic insufficiency can be significantly reduced with intraoperative ICG angiography. The method is also useful in other areas of GI surgery: visualization of lymphatic vessels and lymph nodes, identification of the ureter and urethra, etc. During the search for peritoneal metastases, for example, in 1/3 of the cases, the surgical strategy was changed with the use of ICG [9]. The SENORITA study studied the use of ICG in early gastric cancers. With the help of the dye, they tried to find the sentinel lymph nodes affected by the tumor, and if they were not found, only a stomach-preserving resection was performed, as opposed to the traditional gastrectomy and complete lymph node dissection. Naturally, the lower surgical burden also resulted in a lower complication rate [10].

Wishart and his group used ICG to detect sentinel lymph nodes in breast tumor surgeries, with 100% efficiency [11]. The reliability of the method is clear, and it is also suitable for replacing radioisotopes that are more dangerous from a work safety point of view [12].

In addition to general surgical use, ICG has also been shown to be useful in other manual professions. In neurosurgery, operations can be made more precise by detecting circulation quality. Korja and his colleagues used ICG angiography to examine the circulation of the posterior inferior cerebellar artery during a cerebellar bypass operation and thus performed the operation with greater accuracy [13]. In pediatric heart surgery, Kogon and his colleagues successfully used ICG angiography in coronary reimplantations, surgical solutions for coarctations, and pulmonary artery reconstructions for congenital shunts [14]. Adult cardio-thoracic surgery also uses the method in coronary circulation and myocardium perfusion tests [15].

One of the major barriers to the use of ICG systems is the price. In addition to the large companies, several development groups have created their own tools, the price and quality of which vary widely. Okusanya’s American team has also built an imaging system suitable for detection based on similar principles. The disadvantage of the device is that it is less portable due to it’s size and the cost is $3200 [16]. At this price, professional tools are already available on the market. Already in 2011, Fujisawa’s group presented a self-developed device used in practice (in plastic surgery), the mechanism of which is also similar to our camera [17]. In this case too, the significant cost ($1,600) should be highlighted.

Of course, our own equipment also has it’s limitations. Our primary goal was to be able to produce a practical device as cheaply as possible. Accordingly, the image resolution is not of the best quality, which is especially evident during image recording. An additional requirement would be that the device should also be used for laparoscopic procedures. The first problem can be easily fixed by installing a better-quality camera. The laparoscopic modification is more complicated and requires longer development, but it is included in our plans.

## Conclusion

The possibilities of using ICG and the convincing results are endless. In summary, it can be said that there are many fields of application, from everyday surgical operations to more sophisticated interventions. Its use is becoming more and more widespread in surgical institutes, so there is an increasing demand for simple and inexpensive detector systems to be brought to the market. Our own experiment is proof that even on a very low budget it is possible to create a device that can be used day-to-day. We continue development the with the goal to increase the precision and capabilities of our device, while maintaining the tight budget. After completion of the prototype, we are now exploring the possibility of commercial production.

## Data Availability

The research does not contain any data series relevant to this part. If further information is required, it can be obtained from the corresponding author upon reasonable request.
